# Assessment of the accuracy of ABC/2 variations in traumatic epidural hematoma volume estimation: a retrospective study

**DOI:** 10.7717/peerj.1921

**Published:** 2016-04-11

**Authors:** Pengfei Yan, Ling Yan, Tingting Hu, Zhen Zhang, Jun Feng, Hongyang Zhao

**Affiliations:** 1 Department of Neurosurgery, Union Hospital, Tongji Medical College, Huazhong University of Science and Technology, Wuhan, China; 2 Department of Computer Science, University of Northern BC, Prince George, Canada; 3 Department of Radiology, Union Hospital, Tongji Medical College, Huazhong University of Science and Technology, Wuhan, China

**Keywords:** Epidural hematoma, Volume estimation, Medical image analysis, Automatic segmentation, Stereology

## Abstract

**Background.** The traumatic epidural hematoma (tEDH) volume is often used to assist in tEDH treatment planning and outcome prediction. ABC/2 is a well-accepted volume estimation method that can be used for tEDH volume estimation. Previous studies have proposed different variations of ABC/2; however, it is unclear which variation will provide a higher accuracy. Given the promising clinical contribution of accurate tEDH volume estimations, we sought to assess the accuracy of several ABC/2 variations in tEDH volume estimation.

**Methods.** The study group comprised 53 patients with tEDH who had undergone non-contrast head computed tomography scans. For each patient, the tEDH volume was automatically estimated by eight ABC/2 variations (four traditional and four newly derived) with an in-house program, and results were compared to those from manual planimetry. Linear regression, the closest value, percentage deviation, and Bland-Altman plot were adopted to comprehensively assess accuracy.

**Results.** Among all ABC/2 variations assessed, the traditional variations *y* = 0.5 × *A*_1_*B*_1_*C*_1_ (or *A*_2_*B*_2_*C*_1_) and the newly derived variations *y* = 0.65 × *A*_1_*B*_1_*C*_1_ (or *A*_2_*B*_2_*C*_1_) achieved higher accuracy than the other variations. No significant differences were observed between the estimated volume values generated by these variations and those of planimetry (*p* > 0.05). Comparatively, the former performed better than the latter in general, with smaller mean percentage deviations (7.28 ± 5.90% and 6.42 ± 5.74% versus 19.12 ± 6.33% and 21.28 ± 6.80%, respectively) and more values closest to planimetry (18/53 and 18/53 versus 2/53 and 0/53, respectively). Besides, deviations of most cases in the former fell within the range of <10% (71.70% and 84.91%, respectively), whereas deviations of most cases in the latter were in the range of 10–20% and >20% (90.57% and 96.23, respectively).

**Discussion.** In the current study, we adopted an automatic approach to assess the accuracy of several ABC/2 variations for tEDH volume estimation. Our initial results showed that the variations *y* = 0.5 × *A*_1_*B*_1_*C*_1_ (or *A*_2_*B*_2_*C*_1_) performed better than the other traditional variations, suggesting that the adjusted depth is favorable. In addition, linear regression has been shown to be useful for improving the estimation accuracy of the ABC/2 method, and future studies are warranted to investigate the applicability of such linear regression-derived formulas for clinical application.

## Introduction

Traumatic epidural hematoma (tEDH) is commonly seen in the neurology/neurosurgery department and it is associated with a high morbidity and mortality. The effect of timely surgical evacuation is beneficial in general. Aside from patients’ clinical status and degree of the midline shift, the hematoma volume is a referential parameter in tEDH treatment planning and outcome prediction (Lobato et al., 1998; Jacobs et al., 2011). Therefore, finding a relatively accurate method for tEDH volume estimation would be of clinical interest.

For hematoma volume estimation, a simplified form of the ellipsoid volume equation, commonly denoted as ABC/2, has gained wide acceptance. Assuming the lesion has an ellipsoid shape, the volume can be estimated by measuring three geometrical parameters on neuroradiological images in a few seconds using this method. Previous studies have demonstrated good correlation between the ABC/2 method and the gold-standard planimetry (Sucu, Gokmen & Gelal, 2005; Huttner et al., 2006; Beslow et al., 2010; Kleinman, Hillis & Jordan, 2011; Divani et al., 2011; Hu et al., 2015). In these studies, variations of ABC/2 have been used or proposed. For example, Huttner suggested that ABC/2 should be modified to ABC/3 when estimating oral anticoagulant therapy-associated irregular bleeding volumes (Huttner et al., 2006). However, to our knowledge, it has not been established which variation yields more accurate volume estimations. Clinical decisions to choose one formula over the others are usually arbitrary with the absence of proven references.

In the current study, we selected four ABC/2 variations that are clinically used. First, we assessed the accuracies of these four variations for the tEDH volume estimation, and then we generated and evaluated new variations that may potentially provide a higher accuracy. The advantages and limitations of the ABC/2 method, and possible alternatives to current practice were also discussed.

## Materials & Methods

### Patient selection

This study was approved by the hospital’s institutional review board (no.: WU2015041102). Written informed consent was waived for this low risk, retrospective study. We identified eighty-nine patients diagnosed with tEDH between January 2012 and March 2015. The diagnosis was made by the neuroradiologist on duty, and it was confirmed by one investigator (JF). Thirty-six patients were excluded from this study for at least one of the following reasons: age <18 years, the presence of concurrent adjacent lesions, the presence of isodense tEDHs that are difficult to segment, the existence of image artifacts, and those with computed tomography (CT) scans with a slice thickness >5 mm. Fifty-three patients were finally enrolled in this study. CT images in Digital Imaging and Communications in Medicine (DICOM) format and relevant clinical data were retrieved for each case. Patient information was anonymized and de-identified prior to analysis.

### Measurements

To perform the measurements, after a representative slice was selected, its maximum length(A) was multiplied by the corresponding maximum perpendicular width(B), and then the product was multiplied by the maximum depth of the tEDH to obtain its estimated volume. We assessed both unadjusted and adjusted maximum depths. The unadjusted maximum depth (C_0_) was the slice thickness multiplied by the number of all hematoma-bearing slices. The adjusted maximum depth (C_1_) was the slice thickness multiplied by the adjusted number of hematoma-bearing slices, which was obtained through the following comparison process. For each slice, its hematoma area ratio was defined as the hematoma area of this slice divided by the hematoma area of the representative slice. If the hematoma area ratio was greater than 75%, the particular slice was considered as one hematoma-bearing slice; if the hematoma area ratio was in the range of 25–75%, the particular slice was considered half of a hematoma-bearing slice; and if the hematoma area ratio was less than 25%, the particular slice was not considered a hematoma-bearing slice (Kothari et al., 1996).

Two representative slices were selected for each patient. The first was the slice with the largest hematoma area whose maximum length and width were denoted as A_1_ and B_1_, respectively. The second was the slice in the center whose maximum length and width were denoted as A_2_ and B_2_, respectively; in case of an even number of hematoma-bearing slices, one of the central two slices with a larger hematoma area was chosen (if the hematoma areas of the two slices were the same, then a random slice was chosen). Then we assessed the following four clinically used ABC/2 variations in this study: *A*_1_*B*_1_*C*_0_∕2 (variation 1), *A*_1_*B*_1_*C*_1_∕2 (variation 2), *A*_2_*B*_2_*C*_0_∕2 (variation 3), and *A*_2_*B*_2_*C*_1_∕2 (variation 4).

First, we assessed the estimation accuracy of these four traditional ABC/2 variations compared with gold-standard planimetry; then four corresponding new variations (variation 1′–4′) were created using linear regression analysis and were evaluated.

**Figure 1:**
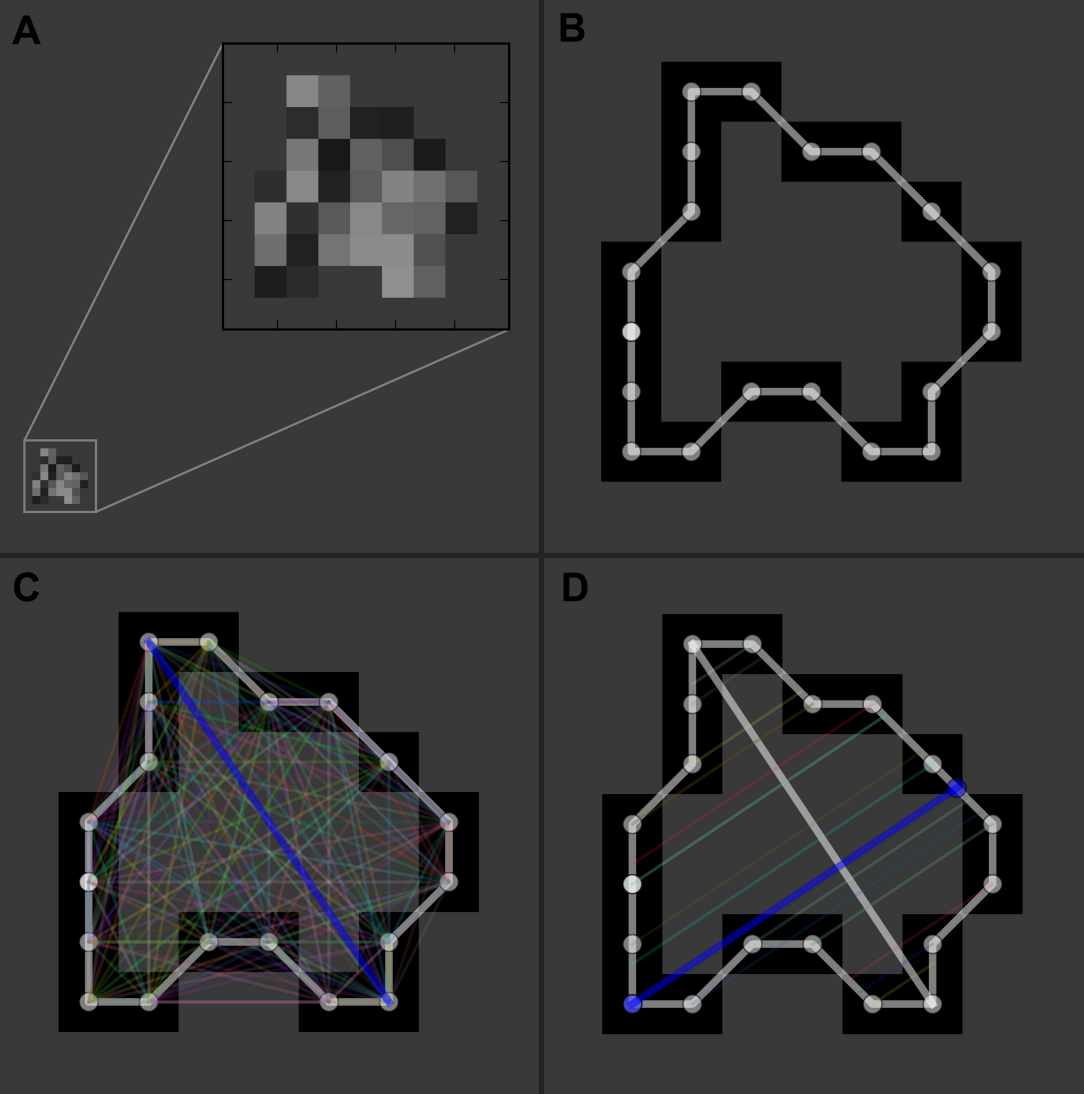
Illustration of the automatic measurement process. For a representative slice (A), the margin of the hematoma was first manually segmented (B); then the distances between any two contour pixels were calculated (C); the two pixels with the longest distance (blue line) determined the maximum length of the hematoma on this slice. The program would then trace along the contour pixels again, and at each pixel, a line was drawn in the direction perpendicular to the maximum length; subsequently, it calculated the distance between the pixel and the intersecting point of the line with the contour (D). After looping over all the contour pixels, distances for each pixel were obtained, and the longest distance (blue line) was used as the maximum width of the hematoma.

Manual segmentation of the hematomas was performed by one investigator (PFY) using ITK-SNAP software (version 3.2.0, University of Pennsylvania) to obtain the aforementioned parameters (Yushkevich et al., 2006). To avoid bias that may be introduced by human raters during the measuring process, we developed a dedicated tool with Python (version 2.7.9; Python Software Foundation) that automatically performs the following tasks: (1) measures the area, maximum length, and maximum width of the hematoma on each CT slice (Fig. 1); and (2) computes values of the different ABC/2 variations for each patient (Suzuki, 1985). Data produced by the program were independently validated by two investigators (LY and PFY). Planimetry was used as the reference standard.

### Statistical analysis

Statistical analysis was performed with the MedCalc package (version 15.4, MedCalc^®^) (Schoonjans et al., 1995). Linear regression was used to generate new ABC/2 variations and determine their correlation with gold-standard planimetry. The closest value was defined as the number of times that one specific variation had a value that was closest to planimetry, and it was regarded as one of the criteria to compare the performance of different ABC/2 variations (Sims et al., 2009). Accuracy was further examined and presented by Bland-Altman plots (Bland & Altman, 1986; Hanneman, 2008). After testing the homogeneity of variance with Levene’s test, the independent t-test was used to analyze differences between estimated values and those of planimetry. A value of *P*≦0.05 was considered statistically significant. If not otherwise stated, categorical values are expressed as numbers with percentages in parentheses; continuous variables are expressed as mean ± standard deviation.

**Figure 2:**
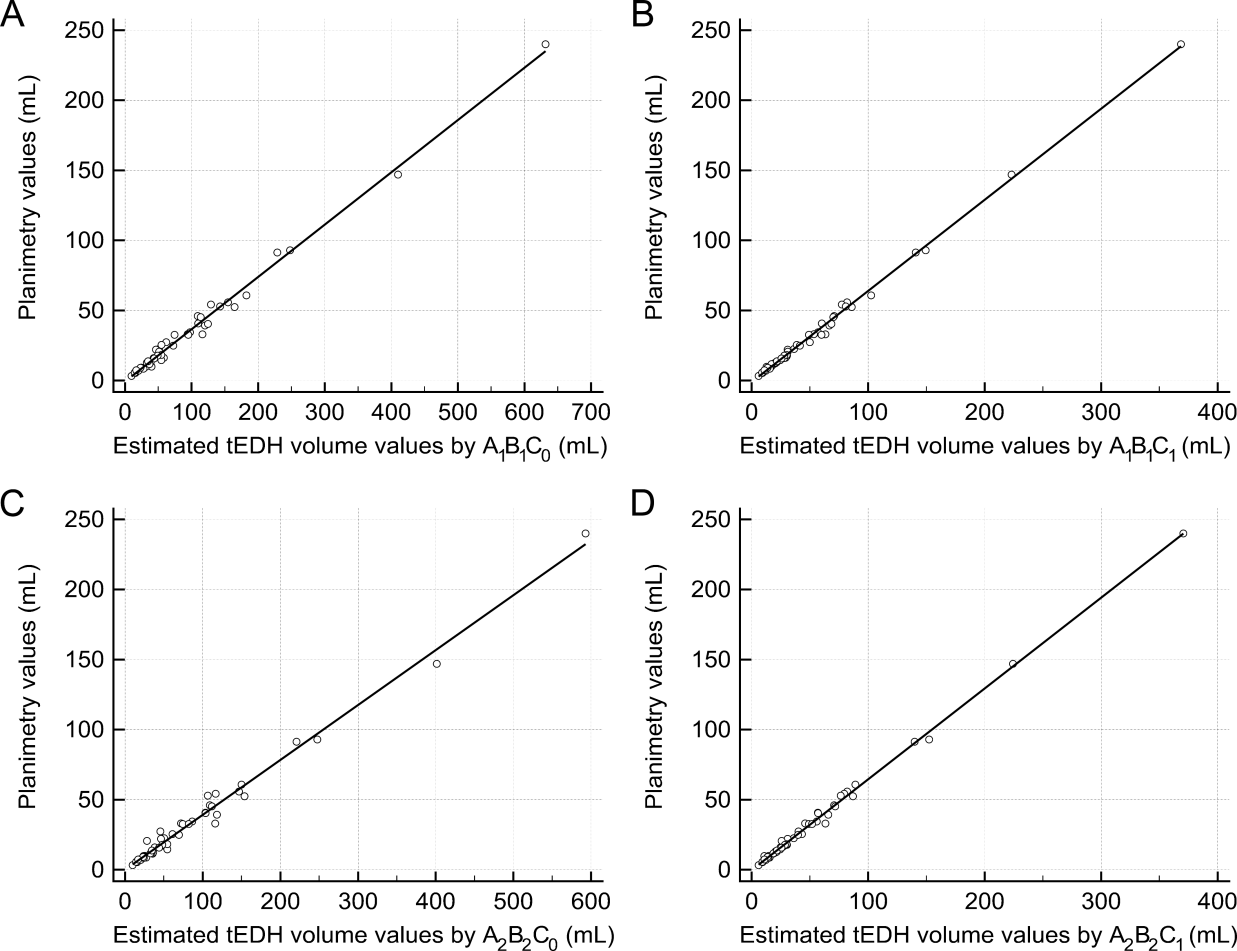
Linear regression analysis. (A) The regression line between the estimated traumatic epidural hematoma (tEDH) volume values by *A*_1_*B*_1_*C*_0_ and planimetry (slope 0.37, intercept −0.75, R^2^ 0.9912) (B) The regression line between the estimated tEDH volume values by *A*_1_*B*_1_*C*_1_ and planimetry (slope 0.65, intercept −1.04, R^2^ 0.9968) (C) The regression line between the estimated tEDH volume values by *A*_2_*B*_2_*C*_0_ and planimetry (slope 0.39, intercept −0.04, R^2^ 0.9869) (D) The regression line between the estimated tEDH volume values by *A*_2_*B*_2_*C*_1_ and planimetry (slope 0.65, intercept −0.17, R^2^ 0.9967).

## Results

Patients’ average age was 41 ± 13 years (range 19–82 years), among which 41 (77.36%) were men and 12 (22.64%) were women. The mean tEDH volume by planimetry was 31.72 ± 39.24 mL (range 3.22–239.98 mL). Table 1 shows the epidural hematoma volume data in detail. Although no statistically significant differences were found between the estimated volume values by the traditional ABC/2 variations and those of planimetry (*p* = 0.19, 0.34, 0.31, and 0.30, respectively; Table 2), variations 2 and 4 generally outperformed variation 1 and 3 as they yielded more values closest to planimetry (30/53 and 10/53, respectively) and had smaller mean percentage deviations (19.12 ± 6.33% and 21.28 ± 6.80%, respectively).

**Table 1:** Epidural hematoma volume data for all 53 patients.

Case No.	Sex	Age	Measured epidural hematoma volume (mL)								
			Planimetry	Variation 1^a^	Variation 2	Variation 3	Variation 4	Variation 1′	Variation 2′	Variation 3′	Variation 4′
1	F	82	9.85	19.84	8.38	12.54	7.38	13.93	9.85	9.74	9.43
2	M	64	60.74	91.14	51.26	74.95	44.50	66.69	65.60	58.42	57.68
3	F	62	52.46	82.27	42.92	76.91	43.47	60.13	54.76	59.95	56.34
4	M	61	3.22	4.91	2.95	4.91	2.95	2.88	2.79	3.79	3.66
5	M	58	34.41	48.77	28.45	43.20	27.90	35.34	35.94	33.66	36.10
6	M	58	17.17	27.39	15.00	24.65	14.08	19.52	18.46	19.19	18.14
7	M	58	17.77	26.35	15.15	26.35	15.15	18.75	18.65	20.51	19.52
8	M	56	6.81	8.58	5.36	8.58	5.36	5.60	5.93	6.65	6.80
9	M	54	11.47	17.80	9.24	17.80	9.24	12.42	10.97	13.84	11.84
10	M	52	5.41	7.81	4.68	7.81	4.68	5.03	5.05	6.05	5.92
11	M	50	7.58	9.73	5.95	9.73	5.95	6.45	6.69	7.55	7.56
12	M	50	12.26	18.04	10.15	18.04	10.15	12.60	12.15	14.03	13.02
13	F	48	27.35	30.91	24.96	22.75	20.12	22.12	31.41	17.70	25.99
14	F	48	22.55	25.41	18.22	25.19	18.06	18.05	22.64	19.61	23.31
15	M	48	7.08	8.34	5.45	8.85	5.79	5.42	6.05	6.86	7.35
16	M	48	8.65	13.59	7.72	13.59	7.72	9.31	9.00	10.56	9.87
17	M	47	16.17	29.07	14.53	26.46	13.23	20.76	17.85	20.60	17.03
18	M	47	9.76	15.60	6.38	11.91	5.41	10.79	7.25	9.25	6.87
19	M	47	33.03	47.02	26.72	36.05	22.94	34.04	33.69	28.08	29.65
20	M	45	91.37	114.31	70.42	110.36	69.96	83.84	90.51	86.04	90.78
21	M	45	25.46	27.34	19.36	30.49	21.60	19.48	24.13	23.74	27.91
22	M	45	46.04	54.53	35.35	54.53	35.35	39.61	44.91	42.50	45.78
23	M	45	13.18	19.30	10.81	17.13	10.28	13.53	13.01	13.33	13.20
24	M	43	55.81	77.30	40.92	73.39	41.01	56.45	52.16	57.21	53.15
25	M	43	32.63	37.19	24.57	37.14	24.54	26.77	30.91	28.93	31.73
26	F	43	7.01	10.06	6.11	10.06	6.11	6.70	6.90	7.81	7.77
27	F	42	33.01	58.06	31.67	58.06	31.67	42.21	40.13	45.24	41.00
28	M	42	39.26	59.97	33.32	59.18	32.88	43.63	42.27	46.12	42.57
29	M	41	9.21	12.04	6.83	12.04	6.83	8.16	7.83	9.35	8.70
30	M	40	14.47	27.18	12.23	27.18	12.23	19.37	14.86	21.16	15.73
31	M	37	32.62	47.44	29.96	40.97	25.87	34.35	37.91	31.91	33.47
32	M	37	12.72	16.36	10.11	17.25	10.65	11.36	12.10	13.42	13.68
33	M	36	11.65	18.29	8.49	16.29	9.31	12.78	10.00	12.67	11.93
34	M	35	239.98	315.83	184.23	296.42	185.26	232.96	238.46	231.17	240.67
35	M	35	22.10	23.14	15.43	23.14	15.43	16.37	19.02	18.01	19.89
36	M	34	54.27	64.49	38.69	58.37	39.69	46.97	49.26	45.49	51.43
37	M	33	40.70	54.88	30.13	51.73	28.40	39.86	38.13	40.31	36.75
38	M	32	8.42	13.81	7.77	12.10	7.18	9.47	9.06	9.40	9.17
39	M	30	24.90	36.22	20.70	34.74	19.85	26.06	25.87	27.06	25.64
40	M	30	40.44	62.27	34.25	51.81	28.50	45.33	43.48	40.37	36.88
41	M	28	92.91	123.92	74.67	123.80	76.18	90.95	96.03	96.52	98.87
42	M	27	15.94	21.40	13.91	19.06	12.39	15.09	17.04	14.82	15.93
43	M	26	5.60	7.26	4.48	7.09	4.59	4.62	4.79	5.49	5.80
44	F	26	20.54	25.59	15.49	14.14	12.83	18.18	19.09	10.99	16.52
45	F	25	9.08	11.60	6.82	11.60	6.82	7.83	7.83	9.01	8.70
46	F	25	18.27	27.21	15.04	27.21	15.04	19.39	18.51	21.18	19.38
47	M	25	17.81	24.55	14.03	23.93	14.24	17.42	17.20	18.62	18.34
48	M	23	52.86	71.34	40.36	53.31	38.23	52.04	51.43	41.54	49.53
49	M	23	146.92	204.97	111.53	200.68	112.14	150.93	143.95	156.49	145.61
50	F	22	45.23	56.78	35.01	55.90	35.87	41.26	44.48	43.56	46.46
51	F	22	13.71	17.29	10.81	17.29	10.81	12.04	13.01	13.45	13.88
52	M	19	15.71	21.88	12.76	21.88	12.76	15.44	15.55	17.02	16.42
53	F	19	7.35	8.41	5.46	8.41	5.46	5.47	6.06	6.52	6.93

**Notes.**

a The eight ABC/2 variations in the table correspond to (1) *A*_1_*B*_1_*C*_0_∕2 (2) *A*_1_*B*_1_*C*_1_∕2 (3) *A*_2_*B*_2_*C*_0_∕2 (4) *A*_2_*B*_2_*C*_1_∕2 (5) 0.37 × *A*_1_*B*_1_*C*_0_ − 0.75 (6) 0.65 × *A*_1_*B*_1_*C*_1_ − 1.04 (7) 0.39 × *A*_2_*B*_2_*C*_0_ − 0.04 (8) 0.65 × *A*_2_*B*_2_*C*_1_ − 0.17, respectively.

**Table 2:** Summary data for the eight ABC/2 variations assessed.

Variation	Formula	Estimated volume (mL)	*P* value^a^	Closest value^b^		Deviations			
				Intra-group	Overall	<10%	10–20%	>20%	Overall (%)
1	0.5 × *A*_1_*B*_1_*C*_0_	43.49 ± 52.34 (4.91 to 315.83)	0.19	6/53	1/53	2 (3.77%)	7 (13.21%)	44 (83.02%)	40.29 ± 19.96 (4.72 to 101.38)
2	0.5 × *A*_1_*B*_1_*C*_1_	25.19 ± 30.13 (2.95 to 184.23)	0.34	30/53	2/53	5 (9.43%)	24 (45.28%)	24 (45.28%)	19.12 ± 6.33 (4.08 to 34.61)
3	0.5 × *A*_2_*B*_2_*C*_0_	40.51 ± 49.73 (4.91 to 296.42)	0.31	7/53	1/53	4 (7.55%)	7 (13.21%)	42 (79.25%)	32.73 ± 17.04 (0.84 to 87.81)
4	0.5 × *A*_2_*B*_2_*C*_1_	24.61 ± 30.23 (2.95 to 185.26)	0.30	10/53	0/53	2 (3.77%)	21 (39.62%)	30 (56.60%)	21.28 ± 6.80 (4.08 to 44.51)
1′	0.37 × *A*_1_*B*_1_*C*_0_ − 0.75	31.43 ± 38.73 (2.88 to 232.96)	0.97	5/53	3/53	25 (47.17%)	20 (37.74%)	8 (15.09%)	11.84 ± 9.04 (1.15 to 41.41)
2′	0.65 × *A*_1_*B*_1_*C*_1_ − 1.04	31.71 ± 39.17 (2.79 to 238.46)	0.99	19/53	18/53	38 (71.70%)	13 (24.53%)	2 (3.77%)	7.28 ± 5.90 (0.02 to 25.66)
3′	0.39 × *A*_2_*B*_2_*C*_0_ − 0.04	31.56 ± 38.79 (3.79 to 231.17)	0.98	10/53	10/53	29 (54.72%)	16 (30.19%)	8 (15.09%)	11.24 ± 11.00 (0.16 to 46.50)
4′	0.65 × *A*_2_*B*_2_*C*_1_ − 0.17	31.82 ± 39.30 (3.66 to 240.67)	0.99	19/53	18/53	45 (84.91%)	6 (11.32%)	2 (3.77%)	6.42 ± 5.74 (0.03 to 29.61)

**Notes.**

a *P*-values were calculated using the independent t-test after testing the homogeneity of variance of the data with Levene’s test.

b The closest value was defined as the number of times that one specific variation had the value that was closest to planimetry.

The four corresponding new variations were as follows: *y* = 0.37 × *A*_1_*B*_1_*C*_0_ − 0.75, *y* = 0.65 × *A*_1_*B*_1_*C*_1_ − 1.04, *y* = 0.39 × *A*_2_*B*_2_*C*_0_ − 0.04, and *y* = 0.65 × *A*_2_*B*_2_*C*_1_ − 0.17 (denoted as variation 1′–4′, respectively; Fig. 2). Still, there were no significant differences in the volume values among these four new variations and planimetry (*p* = 0.97, 0.99, 0.98, and 0.99, respectively). Of the four new variations, however, variations 2′ and 4′ seemed superior to the other two variations: both had higher R^2^ (0.9968 and 0.9967, respectively), smaller mean percentage deviations (7.28 ± 5.90% and 6.42 ± 5.74%, respectively), and more values closest to planimetry (19/53 and 19/53, respectively).

**Figure 3:**
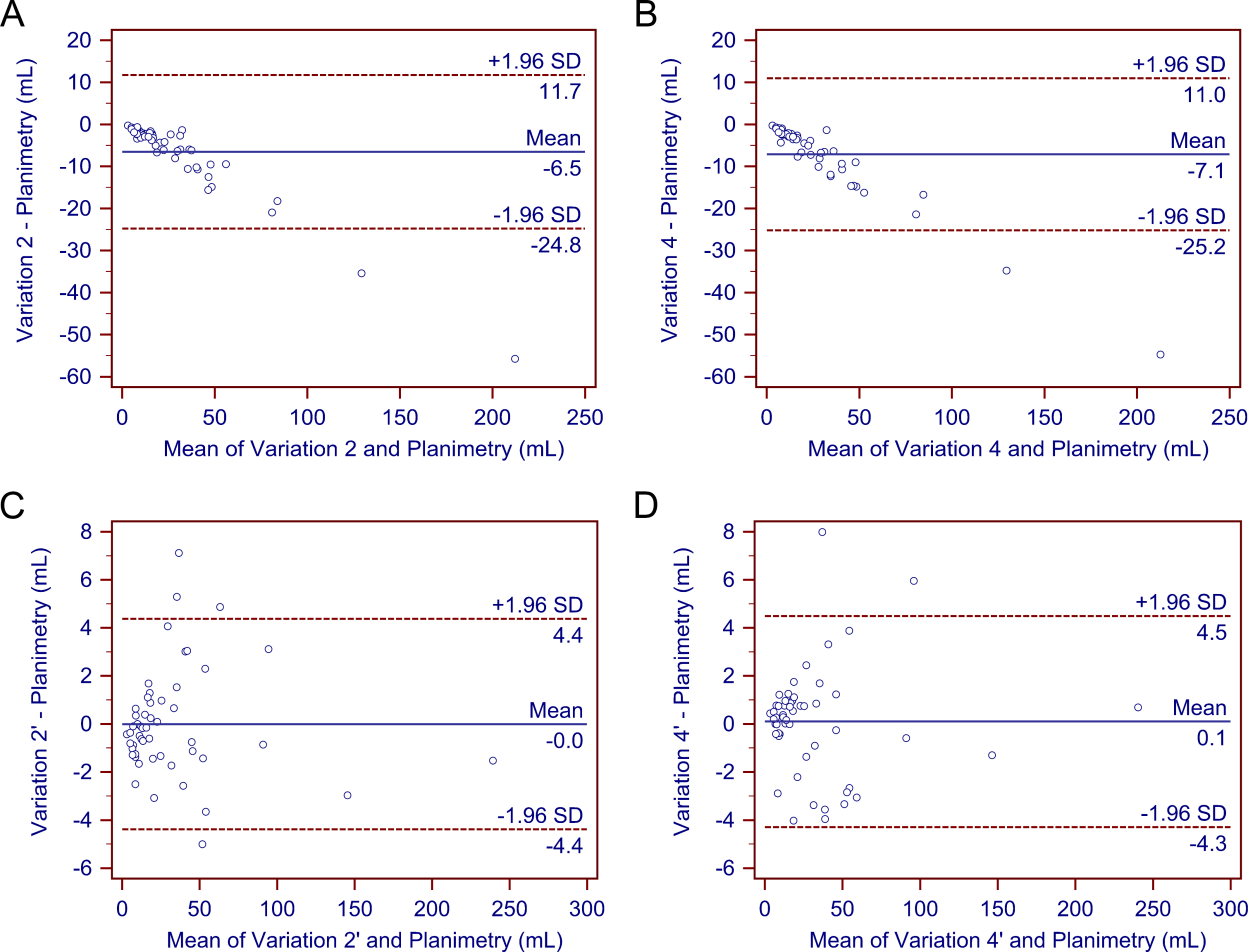
Bland–Altman plots. The Bland–Altman plots demonstrate no significant differences in the estimated traumatic epidural hematoma volume values by variations 2, 4, 2’, and 4’ compared to planimetry. As (A)–(D) show, most of the estimated values are within the limits of the 95% confidence interval.

After determining that variations 2, 4, 2′, and 4′ demonstrated a better performance than the other variations, we further compared these four methods. As previously stated, none of the four methods differed significantly from gold-standard planimetry. Bland-Altman plots confirmed their generally good performance (Fig. 3). Further comparison showed that variations 2’ and 4’ produced more values closest to planimetry (18/53 and 18/53, respectively). They also had smaller mean percentage deviations compared with variations 2 and 4. In addition, deviations of most cases in variations 2’ and 4’ fell within the range of <10% (71.70% and 84.91%, respectively), whereas deviations of most cases in variations 2 and 4 were in the range of 10–20% and >20% (90.57% and 96.23%, respectively). Therefore, variations 2’ and 4’ may be better able to provide a higher accuracy.

## Discussion

The ABC/2 method has gained wide acceptance in volume estimation, and although different variations have been used or proposed in the literature, no uniform variation has been agreed on. For instance, when measuring the length and width of lesions, Kothari et al. (1996) and Sims et al. (2009) chose the slice with the largest hematoma area, which corresponds to A_1_ and B_1_ in our study, whereas Gebel et al. (1998) used the central slice, which corresponds to A_2_ and B_2_ in our study. Likely, when calculating the depth of lesions, Kothari et al. used adjusted values, which corresponds to C_1_ in our study, whereas Sims et al. and Gebel et al. used unadjusted values, which corresponds to C_0_ in our study. Limited data are available regarding which variation has a higher accuracy.

In our study, we first assessed the performance of four clinically used ABC/2 variations for tEDH volume estimation. Our analysis suggested that *y* = 0.5 × *A*_1_*B*_1_*C*_1_ (variation 2) and *y* = 0.5 × *A*_2_*B*_2_*C*_1_ (variation 4) achieved a better performance than the other two variations; comparatively, they seem to be better options for clinical use. Using linear regression analysis, we further attempted to generate and evaluate four corresponding new variations. Of these four new variations, *y* = 0.65 × *A*_1_*B*_1_*C*_1_ − 1.04 (variation 2’) and *y* = 0.65 × *A*_2_*B*_2_*C*_1_ − 0.17 (variation 4’) provided a higher accuracy than the other two variations. In addition, as expected, the general performance of variations 2’ and 4’ were better than that of variations 2 and 4. Hence, they may provide a new perspective on accurate volume estimation. As variations 2’ and 4’ had intercepts close to zero (−1.04 and −0.17, respectively), they could be approximately simplified to *y* = 0.65 × *A*_1_*B*_1_*C*_1_ (or *A*_2_*B*_2_*C*_1_) for calculation convenience.

As described previously, the major difference between variations 2 and 4 (and variations 2’ and 4’) rooted from the selection of the representative slice: the former used the slice with the largest hematoma area as the representative slice, whereas the latter used the central hematoma-bearing slice. Since the performance of both variations seemed to have an equivalent accuracy and the measuring time spent on either representative slice would not differ much, choosing either approach should not result in a noticeable difference. In practical use, determing the slice with the largest hematoma area is a subjective process, thus estimations based on such subjectiveness tend to be unstable; whereas determining the central hematoma-bearing slice is relatively straightforward. The adjusted maximum lesion depth was first proposed by Kothari et al. (1996). No reported studies have specifically compared its estimation accuracy with that of the unadjusted, although the former theoretically seems to be a better option. Interestingly, all the four superior variations in our study (2, 4, 2’, and 4’) incorporated the parameter C_1_ instead of C_0_. This may suggest that the adjusted maximum depth would be more appropriate to use than the unadjusted depth when performing such measurements, at least in patients with tEDH.

Hematoma volume is considered a major factor in the treatment planning in patients with tEDH. According to evidence-based guidelines, patient with tEDH with an hematoma volume >30 mL should undergo surgical evacuation regardless of the patient’s Glasgow Coma Scale (GCS) score; and an epidural hematoma <30 mL with a thickness <15 mm and a midline shift <5 mm in patients with a GCS score >8 without focal deficit can be managed non-surgically (Bullock et al., 2006). Inaccurate calculation of the tEDH volume may possibly lead to either unnecessary surgical procedures or a delay in proper evacuation. In addition, the tEDH volume has also been related to clinical outcome. For instance, in a series of 200 patients with acute epidural hematoma that were surgically treated, Lee et al. (1998) found that a hematoma volume >50 mL was significantly associated with a higher mortality and unfavorable functional outcome. Thus, an accurate estimation of the hematoma volume is clinically important.

The advantages of the ABC/2 method are obvious: it is a bedside method applicable to a clinical scenario, it is time efficient, and the underlying logic is intuitive. However, we noticed one major drawback in the original form of the ABC/2. The original ABC/2 form, i.e., *y* = 0.5 × *ABC*, relies heavily on the assumption that an ellipsoid shape will accurately characterize hematomas; however, in reality many such lesions have irregular shapes (Sorensen et al., 2001). A preliminary categorization of the shape of hematoma before performing the actual measurement theoretically may help improve the estimation accuracy. For example, it may be beneficial if this method is applied only when the hematoma shape is considered ellipsoid-like. However, as there are no clear criteria to categorize hematoma shapes in clinical practice, the effect of such a preliminary shape categorization remains unclear. In contrast, adjusting the original ABC/2 formula seems favorable.

In the present study, we used linear regression analysis and obtained four new ABC/2 variations. These variations were based on clinical data as well as an ellipsoid volume equation; thus, they theoretically should be capable of achieving higher accuracies, and this was proved by our statistical analysis. As these modified methods can provide relatively more accurate volume estimation, adopting this form of methods may help to enhance patient management (e.g., deciding whether a patient should be treated surgically or non-surgically) and outcome prediction. It should be noted, however, that these new variations are yet to apply clinically. The main reason is the limited number of patients enrolled in this study. The new variations were derived from image data of 53 patients with tEDH, which is not a big sample size to make a statement that would commonly apply to clinical settings in general; the formula/coefficient probably would change given a different sample size. Since most tEDHs are biconvex-shaped, we might expect that when the patient population is large enough, the limit of this coefficient may most likely fall somewhere close, so it would be of great interest to expand this study to a large patient group to determine the most appropriate coefficient, which may be recommended for general clinical use. In this study, other than recommending a constant coefficient, we demonstrated the possibility of applying linear regression to improve the estimation accuracy of the ABC/2 method.

Aside from the hematoma shape, the hematoma size is another underlying accuracy-influencing factor (Wang et al., 2009), which may demonstrate its impact in two manners. When a hematoma is small, measuring the length and width of a specific slice becomes difficult and error-prone, resulting in inaccurate volume estimation in these cases. When hematomas are large, the influence of such measurement errors is trivial, and the primary source of estimation inaccuracy becomes the inherent limitation of the ABC/2 method (i.e., differences in the volume between ideal ellipsoid shapes under assumption and the actual hematomas). In the latter case, a positive correlation should exist between the hematoma size and estimation inaccuracy.

It was clear that in a few cases, estimation deviations by variations 2’ and 4’ (*n* = 2 and 2, respectively) reached 20%. The clinical impacts of such relatively large deviations vary among specific situations. Although these cases are uncommon, clinicians should still be aware of possible extreme values that may be produced.

**Figure 4:**
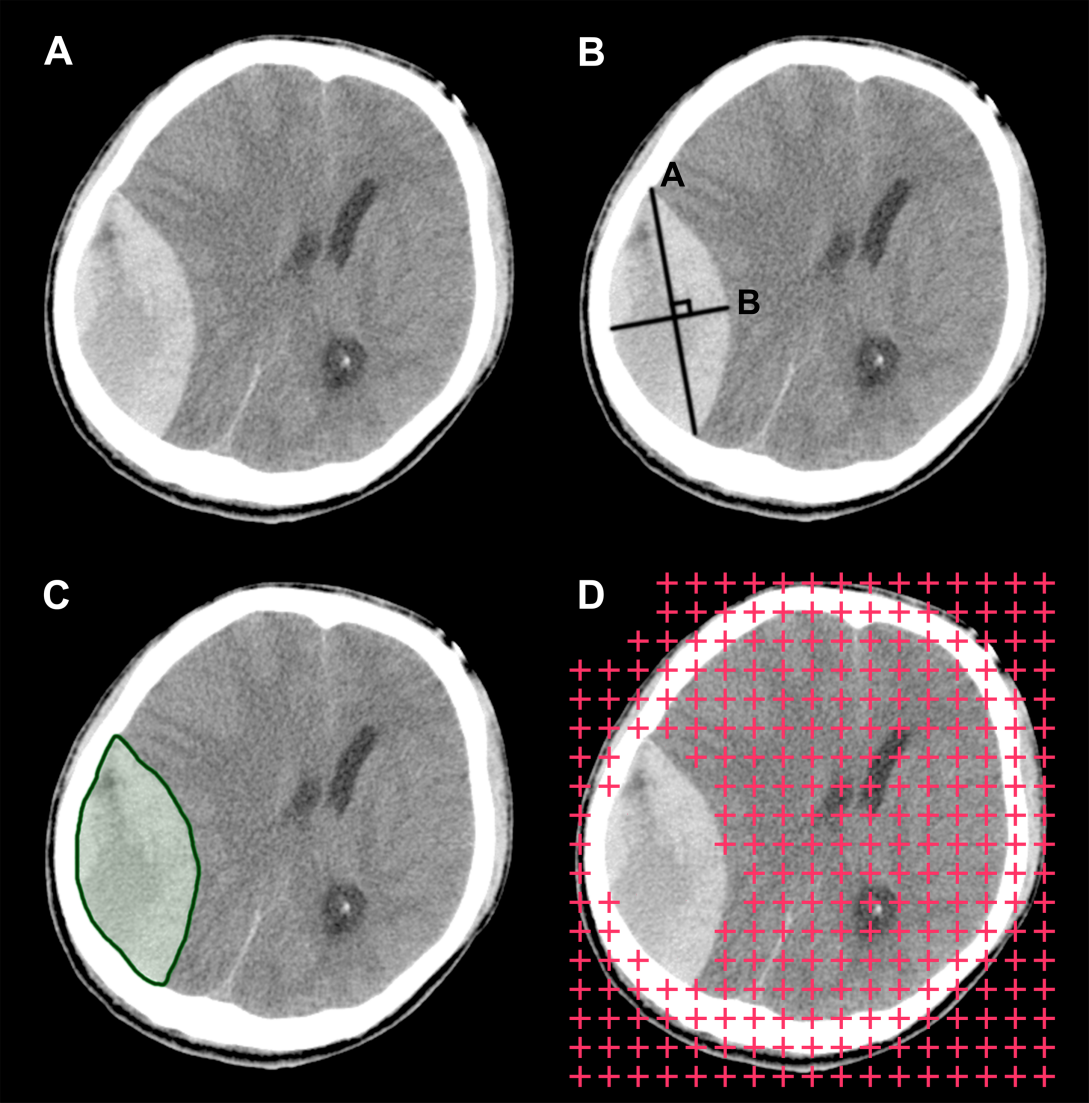
Illustration of the three hematoma volume measurement methods. (A) The original computed tomography scan slice for illustrative purposes. (B) The ABC/2 method. (C) Automatic segmentation. (D) The stereological method or point-counting method.

Two aspects of the design of this study may merit some explanations. First, ABC/2 is commonly considered a method with a high intra- and inter-rater reliability; however, in any research involving a manual process, measurement bias will undoubtedly be introduced by human raters during the measurement process, which would confound the assessment. To help attenuate the bias caused by a manual process, we adopted an automatic approach to perform the measurements. Moreover, although planimetry has been used as the reference standard in related studies, we should keep in mind that in reality it is also just one form of estimation of the actual volume. Planimetry results can be influenced by certain factors such as slice thickness, window/level settings and segmentation inaccuracy. Therefore, various degrees of bias exist when using it as the standard with which to compare the estimation results. However, as currently it is not practical to measure the actual hematoma volume *in vivo*, planimetry remains an appropriate option to use as the reference.

Aside from the ABC/2 method, there are other volume estimation methods worth considering (Fig. 4). One is automatic segmentation (Yushkevich et al., 2006; Whthey & Koles, 2008). Theoretically, programs can identify and segment margins of lesions without human interference, and when segmentation is complete, measuring the lesions’ morphological features, including their volume, is straightforward. Research in this direction is underway. For example, software programs such as ITK-SNAP have implemented the function of semi-automatic segmentation, with which segmentation can be completed in a few minutes for many lesions. Another viable option is the stereological method. Stereology is a technique that concerns the estimation of quantitative three-dimensional morphological data from two-dimensional measurements. It has been widely used on CT and magnetic resonance imaging (MRI) scans, and it has shown good performance on volume estimation of normal intracranial structures as well as tumors (Keller & Roberts, 2009; Sonmez et al., 2010). One additional benefit of this technique is that it can be performed directly on plain CT/MRI films, which is beneficial in emergency settings or when digital DICOM images are unavailable. Future studies are needed to develop and validate these techniques on volume estimation of hematomas. In subsequent studies, we plan to investigate the possibility of combining these two techniques into one integrated process and implement it into a framework. If this method is effective, we believe it would benefit clinical practice to some degree.

## Conclusions

In the current study, we adopted an automatic approach to assess the accuracy of several ABC/2 variations for tEDH volume estimation. Our initial results suggest that the variation *y* = 0.5 × *A*_1_*B*_1_*C*_1_ (or *A*_2_*B*_2_*C*_1_) performed better than the other traditional variations, suggesting that the adjusted depth is favorable. In addition, linear regression has been shown to be useful for improving the estimation accuracy of the ABC/2 method, and future studies using larger sample sizes are warranted to investigate the applicability of such linear regression-derived formulas for clinical application.

## Supplemental Information

10.7717/peerj.1921/supp-1 Supplemental Information 1 Raw data Raw data

## Additional Information and Declarations

### Data Availability

The following information was supplied regarding data availability: Raw data has been uploaded as Supplemental Information.
